# Long-wavelength photoremovable protecting groups: On the way to *in vivo* application

**DOI:** 10.1016/j.csbj.2019.11.007

**Published:** 2019-11-30

**Authors:** Aleksey Yu. Vorobev, Alexander E. Moskalensky

**Affiliations:** a N.N. Vorozhtsov Novosibirsk Institute of Organic Chemistry SB RAS, 9 Lavrentiev Ave., Novosibirsk 630090, Russia; b Novosibirsk State University, Pirogova 2, Novosibirsk 630090, Russia; c Voevodsky Institute of Chemical Kinetics and Combustion SB RAS, Institutskaya str. 3, Novosibirsk 630090, Russia

**Keywords:** photoremovable protecting groups, caged compounds, long-wavelength excitation

## Abstract

Photoremovable protective groups (PPGs) and related “caged” compounds have been recognized as a powerful tool in an arsenal of life science methods. The present review is focused on recent advances in design of “caged” compounds which function in red or near-infrared region. The naive comparison of photon energy with that of organic bond leads to the illusion that long-wavelength activation is possible only for weak chemical bonds like N-N. However, there are different means to overcome this threshold and shift the uncaging functionality into red or near-infrared regions for general organic bonds. We overview these strategies, including the novel photochemical and photophysical mechanisms used in newly developed PPGs, singlet-oxygen-mediated photolysis, and two-photon absorption. Recent advances in science places the infrared-sensitive PPGs to the same usability level as traditional ones, facilitating *in vivo* application of caged compounds.

## 1 Introduction

Light is widely used in biology and medicine for research, diagnostics and therapy [1]. Different approaches are known which use light for the manipulation of biological systems, including optogenetics, photopharmacology [2], light-sensitive liposomes [3] etc. Photoremovable protective groups (PPGs) and related “caged” compounds has been recognized as a powerful tool in an arsenal of life science methods since photolabile cAMP [4] and ATP [5] analogs were introduced. Switching on molecule’s bioactivity by light is an attractive idea in several respects. Firstly, it allows one to activate the molecule *after* the diffusion step and study the unaltered reaction kinetics. Secondly, the spatial resolution of such activation is limited solely by the optical system. It results in new possibilities, for instance, intercellular signaling or impulse propagation can be easily studied. Finally, light can pass through cellular membranes and organelles and thus enables the intracellular control of chemical processes. However, traditionally used PPGs based on *o*-nitrobenzyl, nitroanilide, phenacyl, benzoin, coumarin, *etc.* moieties [6] are sensitive to UV radition (300–370 nm), which is damaging to living cells. In contrast, near-infrared radiation (approximately 700–1100 nm) is not cytotoxic and penetrates much deeper into living tissues. This ability is used in many theranostic applications. *In vivo* uncaging opens a new way in therapy, perhaps complementary to photopharmacology and photodynamic therapy [7].

In the present review, we highlight recent advances in design of “caged” compounds sensitive to red and infrared light. “Caged” compounds are defined as relatively small molecules that can release substance of interest under the action of light. Several approaches to carry this functionality to long-wavelength region are known. However, this problem has no simple solution because typical organic bond dissociation energy is about 350–400 kJ/mol, which corresponds to a 340 nm UV light. Unfortunately, the energy of infrared photon is at least twice less. Therefore, one needs to either use the energy of multiple photons or somehow weaken the bond. Below, we describe several strategies to solve the problem. First section is devoted to a near-infrared PPGs which work through a direct single-photon photoprocess. Second section describes compounds with photorelease step that involves a reaction with singlet oxygen. In the third section, we give a brief overview of two-photon absorbing PPGs. Last section concludes the paper and gives references for further reading on connected topics, including photon upconversion-based photorelease technique.

## 2 Single-photon PPGs

The “uncaging” reaction implies the dissociation of covalent bond between PPG and leaving group (LG) and therefore requires energy. For a rough estimate the average bond energy can be used to calculate the photon wavelength needed for the dissociation. For instance, C-C and C-O bonds correspond to 320–350 nm light, slightly weaker C-N bond – up to 395 nm. This simple consideration shows why the majority of known PPGs works in the near-UV spectral region. However, the process is far from simple “scission” and often proceeds through several stages which typically include intramolecular electron or hydrogen atom transfer, rearrangements or cyclizations, and solvolysis.

**Fig. 1:**
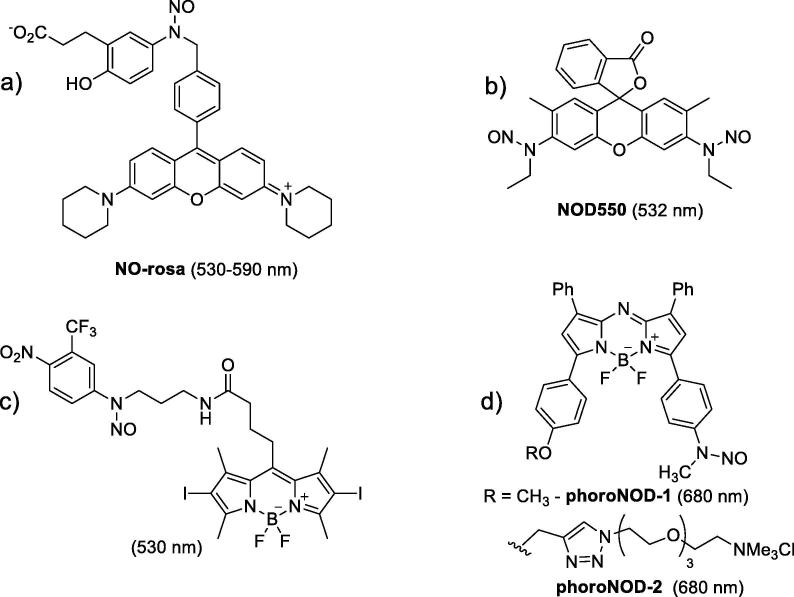
Nitric oxide (NO) donors activated with long-wavelength light. a) **NO-Rosa**[10]; b) **NOD550**[13]; c) **NOBL-1**[16]; d) **photoNOD-1** and **photoNOD-2**, [19]. The uncaging wavelength is shown near each structure.

Interesting example of weak chemical bond, still relevant for biomedical applications, is N-NO. The average energy of dissociation in this case corresponds to ~730 nm wavelength. The nitric oxide produced upon dissociation is of high interest for biological studies [8], so much efforts were made to prepare phototriggered NO-donors [9]. To achieve a cleavage of N-NO, a chromophore with strong absorbance in near-infrared spectral region needs to be attached to this group. For instance, in was shown that the rhodamine moiety enables effective light absorption with electron transfer from N-NO to dye fragment which facilitates N-N bond dissociation. This is the basis of **NO-Rosa** (Fig. 1a) [10] and related compounds [11] which release NO under illumination of 530–590 nm yellow-green light. Light-controlled rat aorta vasolidation with **NO-Rosa** was demonstrated [10]. Besides, perspectives of such NO-donors for erectile dysfunction treatment have been reported [12]. Rhodamine derivatives bearing N-NO fragments attached to xanthene core such as N-nitrosorhodamine 6G (**NOD550)** (Fig. 1b) [13] also possess NO-releasing under green light illumination. As **NOD550** gives highly fluorescent dye upon decomposition, it was used for monitoring mitochondrial dynamics [14]. Another water-soluble rhodamine derivative **NOD565** [15] showed antifungal activity and platelets activation inhibition while irradiated by green light.

BODIPY core represents an attractive chromophore with strong absorption in green region and easily tunable spectral properties. **NOBL-1** derivative was applied for vasodilatation [16] or rat penile corpus cavernosum relaxation under blue light irradiation (470–500 nm). An interesting feature of BODIPY-N-NO hybrid (Fig. 1c) to generate singlet oxygen together with NO was reported [17]. This substance and its photodegradation product were not cytotoxic for normal and cancer cells, but the hybrid caused cancer cell death under irradiation. Similar BODIPY-N-NO hybrid [18] has close properties. It was noted that in all cases energy transfer from dye fragment to N-NO proceeded through electron transfer from N-NO to exited dye moiety.

The application of aza-BODIPY core (Fig. 1d, **photoNOD-1** and **photoNOD-2**) enabled NO release upon single-photon NIR irradiation [19]. Both substances showed high stability toward biological red-ox systems, and showed low cytotoxicity and toxicity. Nitric oxide donor **photoNOD-1** possessed high activity to inhibit tumor growth in mice under regular administration and NIR irradiation.

**Fig. 2:**
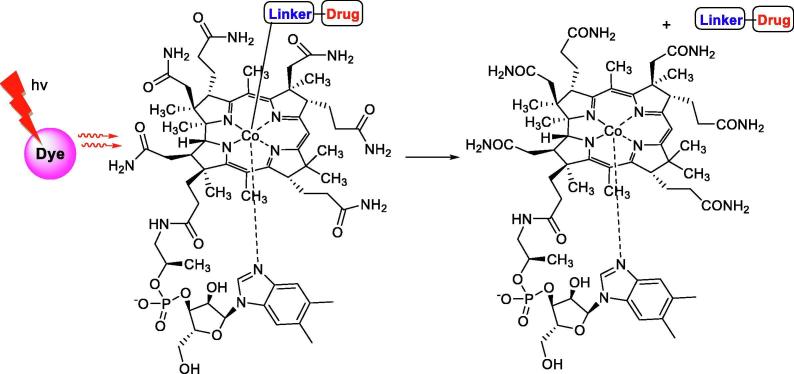
Analogs of vitamin B12 as “caging” groups [20], [21].

Another example of weaker bond recently used for “caging” is carbon-cobalt connection in vitamin B_12_ analogs. The bond energy is approximately 1.3–1.6 eV [22], corresponding to the wavelength up to 950 nm. Although the vitamin B_12_ does not absorb light above 550 nm, it was shown that various cyanine fluorophores can be used as antennas to capture long wavelength light then transfer energy to the “cage” and promote scission of the Co-C bond at wavelengths up to 800 nm (Fig. 2; [20], [21]). This principle has been used for light-triggered anti-inflammatory drug release with use of erythrocytes as carriers.

**Fig. 3:**
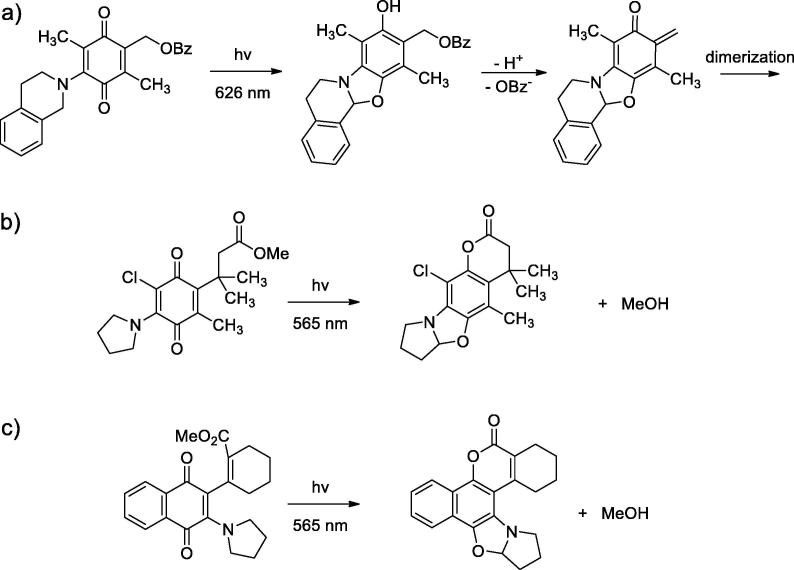
a) Benzoquinone-based photocage undergoes photocyclization and then thermal elimination of benzoic acid [23]; b) A “quinone trimethyl lock” PPG [24]; c) PPG based on *cis*-alkenyl substituted quinones [25].

If the sole photon energy is not sufficient to bond breaking, additional photochemical transformations are essential for uncaging [26]. For instance, benzoquinone-based photocage, first introduced in 2006 [27], undergoes photocyclization at 458 nm and then thermal elimination of LG. Recently, such type of compounds was tuned to 626 nm activation by expanding the amine ring size and rendering the C-H bond benzylic [23] (Fig. 3a). The “quinone trimethyl lock” structures were described recently as general design for long-wavelength photoremovable protecting groups for alcohols and amines [24] (Fig. 3b). They also rely on photocyclization, and the detailed uncaging mechanism was exhaustively described [28]. Analogous process takes place in *cis*-alkenyl substituted quinones (Fig. 3c) [25].

**Fig. 4:**
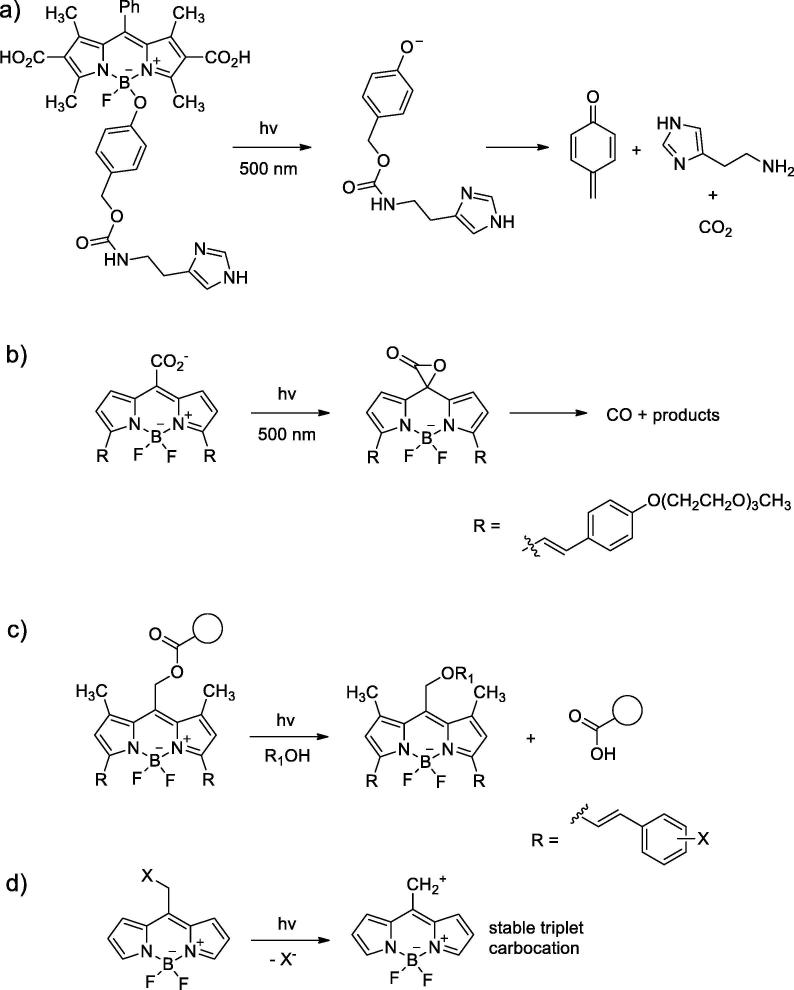
BODIPY-based PPGs a) Group base on B-O bond and the uncaging mechanism; b) substituted BODIPY for CO photorelease; c) *meso*-CH_2_X BODIPY derivatives as near-infrared caging groups; d) proposed scheme of photolysis [32].

Blue-light activated versions of BODIPY-based PPGs for small organic molecules, e.g. histamine, were reported (Fig. 4a) [29]. The uncaging process is based on photodissociation of B-O bond. Several months later, another group reported a near-infrared release of CO by styryl-substituted BODIPY *meso*-carboxylic acids [30]. The reaction proceeds through intramolecular electron transfer from CO_2_^−^ to heterocyclic core with further cyclization and CO extrusion (Fig. 4b). *meso*-CH_2_X BODIPY derivatives were suggested to be promising caging groups. Structure-function relationship for such PPGs was systematically studied, and a strategy to obtain near-infrared caged compounds was proposed and tested [31].

It was shown that extending of the conjugated system (for instance, by additional styryl groups; Fig. 4c) allows one to shift the absorption maximum to longer wavelength, up to 700 nm preserving the uncaging capability. The question remains how far red it can be shifted without significant loss of efficiency. It is remarkable that uncaging launches by single-photon absorption and quantum yield is comparable with traditionally used *o-*nitrobenzyl groups.

The reason of low uncaging energy lies in the photochemistry of such BODIPYs. Experimental results showed the enhancement of uncaging quantum yield by the presence of a heavy atom and reduced efficiency in presence of oxygen. It indicates that photochemical reaction which lead to the dissociation of LG goes through the triplet state [33]. The PPG-LG bond is cleaved by heterolysis to yield the LG^-^ and PPG residual as carbocation (Fig. 4d). Importantly, the carbocation retains triplet character of the whole molecule, and can be viewed as stable diradical in this state [32]. Quantum mechanics calculations for the carbocation in Fig. 3d show that triplet energy level of the carbocation residual is only 1.63 eV, which is comparable to the infrared photon energy at ~760 nm. The triplet energy level of the whole (neutral) molecule is only slightly lower according to calculations (1.36 eV, ~910 nm) or even higher according to experimental estimations [34].

The remarkably small gap might be explained by the conical intersection between molecular triplet and the diradical energy surfaces. It means that the triplet state of the whole caged molecule can degrade to the dissociated state virtually free of energy. In other words, the PPG-LG bond in this state is very weak. The only requirement for the uncaging is to excite the molecule to the triplet state, which can be done through the intersystem crossing from the first singlet excited state. According to the quantum mechanics calculations and fluorescence measurement for the same compound, its energy level is 2.2–2.3 eV, which corresponds to the photon wavelength of 560 nm. While it can be lowered by extending conjugation system, it is still unclear where is the limit of breaking the conical intersection nature of photodissociation.

Other molecular structures with similar properties may exist, and low S_0_–S_1_ energy gap of the residual cation has been suggested as a good predictor that conical intersection nature of photodissociation might take place [32]. Recently, coumarin-based PPG with ability of intramolecular carbocation trapping was introduced [35].

## 3 Singlet oxygen-mediated uncaging

The strategy of so-called self-sensitization is employed in a class of cyanine-based PPGs. It constitutes a formation of singlet oxygen, which then oxidize the molecule and eventually leads to the liberation of LG. Since the singlet oxygen is relatively long-lived molecule, energy of several photons may be effectively stored before the “oxidative attack”.

A disadvantage of this technique is that reactive oxygen species could cause the undesired damage of surrounding tissues. On the other hand, they are able to enhance the action of cytotoxic agents as singlet oxygen is the main effector of photodynamic therapy.

**Fig. 5:**
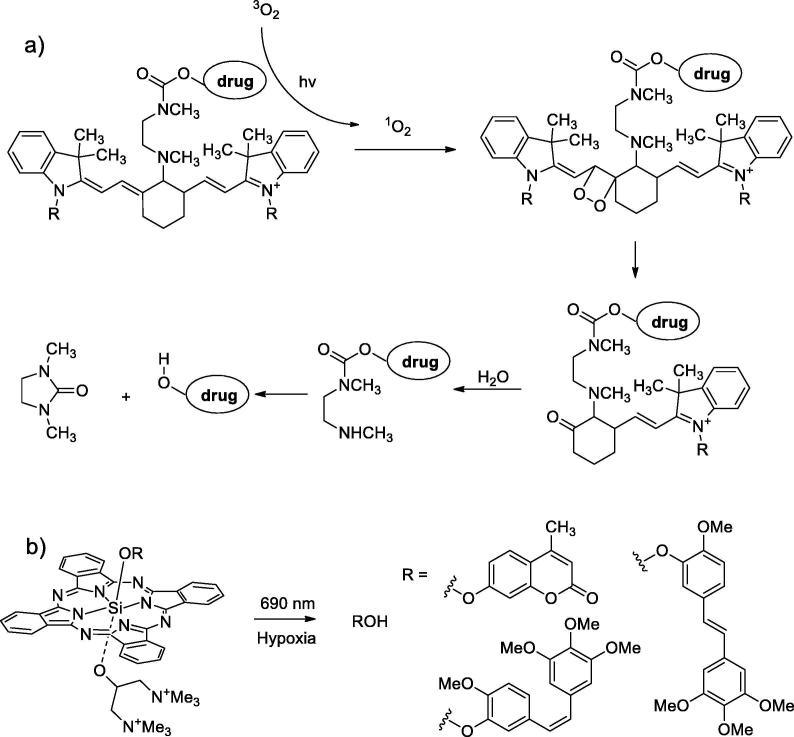
Singlet-oxygen mediated uncaging. a) Cyanine-based caged compound and the photorelease scheme [36]; b) Silicon phthalocyanines releasing phenolic compounds used for tumor targeted treatment [42].

The first example of such PPG was estblished by [36], [37]. Efficient uncaging was reported under 690 nm light, which consists of photooxidative and then hydrolytic steps (Fig. 5a). 4-hydroxycyclofen bearing such caging group showed significant cytotoxicity against breast cancer MCF-7 cells under irradiation. Later this method was extended by two groups to uncage the aryl amines by similar oxidation pathway [38], [39]. Conjugation of cyanine dyes with antibodies and cytotoxic drugs provides unique possibilities to tumor targeted drug release [40], [41].

Silicon phthalocyanines with unsymmetrical axial substitution (Fig. 5b) were found to release phenolic compounds under NIR irradiation in hypoxia conditions, whereas in oxygenated conditions singlet oxygen was produced [42]. This property was used for tumor targeted treatment.

## 4 Two-photon absorption PPGs

Two-photon (and multiphoton) techniques are powerful tools in biomedicine, from microscopy to *in vivo* imaging and therapy. They are based upon simultaneous absorption of two photons. Unfortunately, the probability of this process is quite low. Sufficient photon density is typically obtained using pulsed femtosecond laser source, which limits the applicability to a scientific research in specially equipped laboratories. On one hand, it enables better spatial resolution because is effective only in small focal volume, where the density of photons is high enough. On the other hand, specially designed molecules with significant two-photon absorption cross section are needed. Below, we overview the PPGs which can work in two-photon regime. It retains tissue-penetrating advantages of infrared light activation, and the cumulative energy of two photons is enough to cleave th PPG-LG bond. There are excellent reviews on this topic (see e.g. [43], [44]), so we briefly designate the most widespread two-photon PPGs families and focus on the most recent publications.

**Fig. 6:**
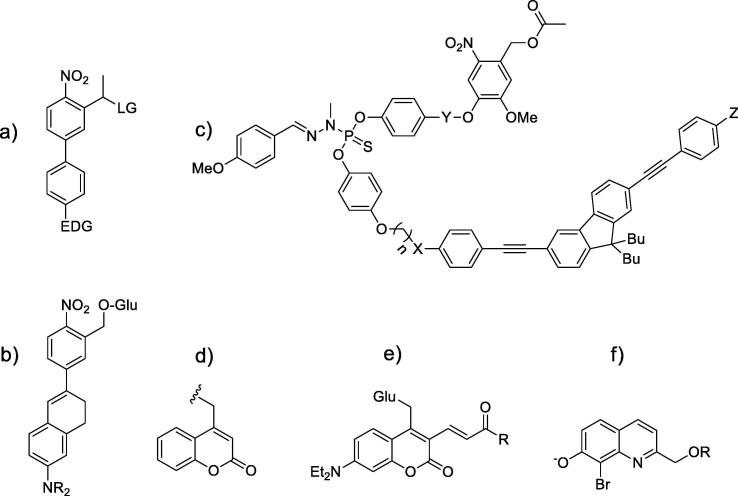
Two-photon absorbing caged compounds a) Biphenyl-based PPGs [48]; b) PPG based on 1,2-dihydronaphthalene structure [49]; c) a two-photon absorbing antenna with *o*-nitrobenzyl- and d) nitroindoline-caged acetic acid [54]; e) Structure of unsubstituted coumarin PPG; f) Structure of unsubstituted quinoline PPG.

The most widespread *o*-nitrobenzyl groups have poor two-photon absorption cross-section [45], although those with 3,4-dimethoxy groups were used, for instance, to induce calcium waves in astrocytes by uncaging of IP3 using 720 nm femtosecond excitation [46]. Further advance was related to conjugation of these groups with two-photon absorbing moieties [47]. The absorbed energy is then transferred to the *o*-nitrobenzyl group to induce uncaging. Perhaps the best examples of this strategy are biphenyl-based PPGs [48] (Fig. 6a), groups based on 1,2-dihydronaphthalene structure [49] (Fig. 6b) and caged calcium chelators [50]. Similar group is incorporated into the newly developed nitrodibenzofuran-based PPGs [51], [52]. Same approach was applied to other caging groups [53]. Fig. 6c shows a molecular dyad described in [54], where the lower part of the molecule serves as two-photon absorbing antenna, which excites usual *o*-nitrobenzyl caging groups (upper part) *via* charge transfer, inducing the release of acetic acid. Recently, compounds based on bisstyrylthiophene (BIST) was developed for photorelease of calcium and GABA [62], [63].

Two-photon PPGs based on coumarin (Fig. 6d) and quinoline (Fig. 6f) dyes are probably the most used. The structure-efficiency relationship for aminoquinoline-based PPG was studied in [55]. Coumarin photochemistry and the influence of different substituents is the area of active research [35], [56]. For instance, coumarin-based cell-permeable caged phosphates were reported that can be photolyzed by 800 nm two-photon photolysis. The derivative of 7-diethylaminocoumarin DEAC450 was used for “caging” of glutamate (Fig. 6e; [57]). It was shown that two-photon excitation at 900 nm at spine heads on pyramidal neurons in acutely isolated brain slices generated postsynaptic responses. Later the efficiency of this compound was improved using systematic approach, including calculations and experimental synthesis [58]. It was shown that subtle tuning of polarization in the ground-state and confinement of the photo-induced intramolecular charge transfer upon excitation is responsible for enhancing two-photon absorption while maintaining large uncaging efficiency.

## 5 Conclusion and outlook

The transfer of the “caged” compounds technique to near-infrared region is the cornerstone for its *in vivo* application. In this review, we highlighted recent papers where long-wavelength uncaging was reported. There are three main strategies used for this purpose. Firstly, several newly developed PPGs possess so little uncaging threshold that infrared photon energy is enough. It, in turn, may be achieved by different means, including the use of weak bonds (e.g. N-NO, Co-C), additional energy release from PPG molecular photorearrangement, and low-lying triplet carbocation state of PPG residual. Secondly, some papers describe singlet-oxygen-mediated photolysis, which relies on photocatalyzed oxidation of “caged” compound by reactive oxygen species eventually leading to the release of LG. Thirdly, molecules with significant two-photon absorption may be activated using near-infrared light. A related technique is photon upconversion, a process where the energy of several photons is used to create one with correspondingly shorter wavelength. In contrast to two-photon techniques, the upconversion does not require enormous light intensities, but specially designed nanoparticles should be used. For instance, upconverting nanoparticles coated by protein kinase A, which was in turn “caged” by UV PPG, was described [59]. It was shown that illumination of nanoparticles by 980 nm laser induced activation of the enzyme. However, this strategy is a bit aside from the present review; interested readers are encouraged to look at other reviews on this topic [60], [61].

## Declaration of Competing Interest

The authors declare that they have no known competing financial interests or personal relationships that could have appeared to influence the work reported in this paper.
